# Decadal Changes in the World's Coastal Latitudinal Temperature Gradients

**DOI:** 10.1371/journal.pone.0067596

**Published:** 2013-06-18

**Authors:** Hannes Baumann, Owen Doherty

**Affiliations:** 1 School of Marine and Atmospheric Sciences, Stony Brook University, Stony Brook, New York, United States of America; 2 Scripps Institution of Oceanography, University of California San Diego, La Jolla, California, United States of America; University of Vigo, Spain

## Abstract

Most of the world's living marine resources inhabit coastal environments, where average thermal conditions change predictably with latitude. These coastal latitudinal temperature gradients (CLTG) coincide with important ecological clines,e.g., in marine species diversity or adaptive genetic variations, but how tightly thermal and ecological gradients are linked remains unclear. A first step is to consistently characterize the world's CLTGs. We extracted coastal cells from a global 1°×1° dataset of weekly sea surface temperatures (SST, 1982–2012) to quantify spatial and temporal variability of the world's 11 major CLTGs. Gradient strength, i.e., the slope of the linear mean-SST/latitude relationship, varied 3-fold between the steepest (North-American Atlantic and Asian Pacific gradients: −0.91°C and −0.68°C lat^−1^, respectively) and weakest CLTGs (African Indian Ocean and the South- and North-American Pacific gradients: −0.28, −0.29, −0.32°C lat^−1^, respectively). Analyzing CLTG strength by year revealed that seven gradients have weakened by 3–10% over the past three decades due to increased warming at high compared to low latitudes. Almost the entire South-American Pacific gradient (6–47°S), however, has considerably cooled over the study period (−0.3 to −1.7°C, 31 years), and the substantial weakening of the North-American Atlantic gradient (−10%) was due to warming at high latitudes (42–60°N, +0.8 to +1.6°C,31 years) and significant mid-latitude cooling (Florida to Cape Hatteras 26–35°N, −0.5 to −2.2°C, 31 years). Average SST trends rarely resulted from uniform shifts throughout the year; instead individual seasonal warming or cooling patterns elicited the observed changes in annual means. This is consistent with our finding of increased seasonality (i.e., summer-winter SST amplitude) in three quarters of all coastal cells (331 of 433). Our study highlights the regionally variable footprint of global climate change, while emphasizing ecological implications of changing CLTGs, which are likely driving observed spatial and temporal clines in coastal marine life.

## Introduction

Coastal and continental shelf regions are vitally important to humans [1]. They constitute the most diverse and productive parts of the world's oceans and provide an estimated 43% of all ecosystem services [2]. Hence, understanding major drivers of change in coastal ecosystems, both in space and time, remains a long-standing scientific goal that has become more urgent due to unfolding global climate change [3]. Given that temperature is the central abiotic variable affecting marine life from the cell to the ecosystem level [4], [5], studies of coastal ecological change are particularly interested in spatial and temporal temperature patterns along coastal areas.

One important large-scale pattern is the common decrease in temperature with latitude. Although ubiquitous on earth, latitudinal temperature gradients differ greatly between terrestrial and marine systems or between the open ocean and coastal areas as a result of regional wind and cloud patterns, alongshore currents and coastal upwelling [6]–[8]. Latitudinal temperature patterns coincide with – and potentially drive – important ecological clines such as the common latitudinal decrease in species diversity [9] or many morphological, physiological and life history traits that are linked directly or indirectly to temperature (e.g., body size [10], [11], growth and development rates [12], life span [13], fecundity [14], recruitment [15], and vertebral number [16], [17]).

However, despite their potential ecological significance, coastal latitudinal temperature gradients (CLTG) are rarely implicated in studies of large-scale ecological change, perhaps because they have yet to be consistently quantified. While temperature patterns and trends in average open oceans have been a scientific focus for decades [7], [18]–[20], the world's coastal temperature patterns have received considerably less attention [21]. Recently, Lima and Wethey [6] used sea surface temperature (SST) data of the highest available spatial and temporal resolution (1/4 degree daily SST analysis) to document the heterogeneity in fine-scale coastal SST changes as well as trends in the number of extreme hot or cold days. Here we explore how regional SST patterns determine the diversity of CLTGs on larger spatial and temporal scales, which is useful for potential analyses of spatial trait patterns in the ocean or ecological consequences of temporal changes over the past three decades.

We used SSTs from a global data set as a proxy for ambient thermal conditions in the coastal waters. Selecting only the coastal cells along the world's major north-south oriented continental coastlines enabled us to define and comparatively characterize 11 CLTGs. Apart from SST itself, our spatio-temporal analyses included a second ecologically relevant variable, seasonality, which describes the magnitude of seasonal temperature fluctuations. For many coastal organisms, seasonality plays an important role in determining the length of growing and spawning seasons [12], the severity of winter mortality [22] or the degree of temperature-dependent sex determination [23].

The two goals of this study were (1) to quantify the regional variability in long-term average strength and seasonality among the world's major CLTGs and (2) to examine how these gradients have themselves changed over the past three decades. Specifically, we hypothesized that greater warming at high compared to low latitudes [3], [6], [20] will lead to a general weakening in gradient strengths, accompanied by trends towards more extreme seasons [24]. For clarity, the scope of these analyses was limited to large scale patterns in space and time as relevant to large-scale ecological clines, while purposely excluding analyses of gradient features on finer spatial and temporal scales.

## Methods

**Figure 1:**
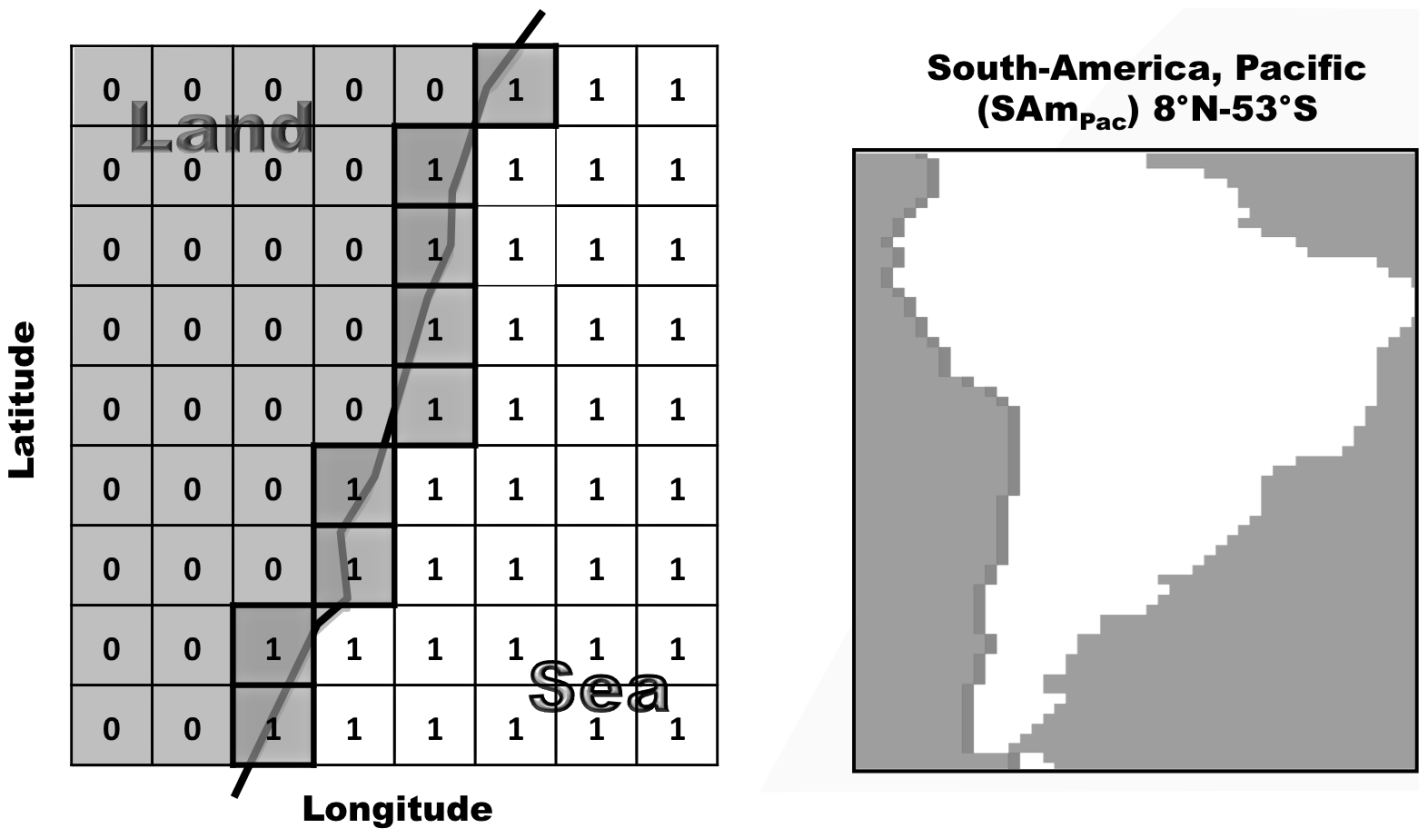
Extracting coastal latitudinal SST gradients. **A**: the 1°×1° land-see mask of the NOAA OISST (V2) dataset was used to select at each latitude and gradient the ‘sea’ cells (“1”, white) closest to land (“0”, dark grey). **B**: example of extracted cells (black) to characterize the South-American Pacific gradient (SAm_Pac_).

CLTGs were extracted from the NOAA Optimum Interpolation (OI) SST product (V2), a 31 year dataset (1982–2012) of global SSTs with a 1°×1° spatial and one week temporal resolution[25]. SSTs comprise satellite AVHRR data (Advanced Very High Resolution Radiometer) blended with *in situ* observations (International Comprehensive Ocean-Atmosphere Data Set, ICOADS) using optimal interpolation algorithms described in Reynolds et al. [26] and Smith et al. [27]. From the global dataset we extracted SSTs only from coastal cells, defined as ‘sea’ cells immediately adjacent to ‘land’ in the datasets land-sea mask (Fig.1a). To be consistent, we excluded major islands from continental coastlines (e.g., British Isles, Japan, Madagascar) and omitted latitudes that would have led to inclusion of large embayments, gulf or marginal seas (e.g., Baltic, Gulf of Mexico, Red Sea,). No analysis was performed over land so that sea cells, even if partially extending over land, only contain SST data. Lima and Wethey [6] demonstrated that the degree of land ‘contamination’ of coastal SST cells did not bias SST trends in a similar dataset. We then further selected only those cells that belonged to the world's major north-south oriented continental coastlines (e.g., Fig.1b), yielding a total of 433 cells from 11 gradients: the North-American Pacific and Atlantic gradients (NAm_Pac_: 27–60°N, NAm_Atl_: 26–60°N), the South-American Pacific and Atlantic gradients (SAm_Pac_: 8°N–55°S, SAm_Atl_: 8°N–55°S), the North- and South-African Atlantic gradients (NAf_Atl_: 5–35°N, SAf_Atl_, 1°N–34°S), the African Indian Ocean gradient (Af_Ind_: 23°N–34°S), the European Atlantic gradient (Eu_Atl_: 37–70°N), the Asian Pacific gradient (As_Pac_: 22–61°N), and the Australian Indian Ocean and Pacific gradients (Au_Ind_: 12–35°S, Au_Pac_: 11–39°S).

To characterize average gradient strengths, we calculated the long-term mean SST for each latitude (31 years ×52 weeks  = 1,612 data points) and used the gradient-specific slope of the linear regression of mean SST against latitude. In addition, we calculated the long-term SST minimum and maximum (average of the 31 annual SST minima and maxima) for each latitude of each CLTG, and used the difference SST_max_ - SST_min_ as a measure of seasonality. To examine how gradient strength changed over time, annual gradient slopes (i.e., from linear regressions of annual mean SSTs against latitude) were linearly regressed against year. The total change in gradient strength was then expressed as the percentage difference between the predicted strength at the beginning (1982) and end (2012) of our 31 year time series. In addition, we characterized strength and temporal variability of partial sections of some gradients, where regional rates of latitudinal SST change differed markedly from the average gradient strength (Table 1).

**Table 1:** Strength and decadal changes of the world's coastal latitudinal temperature gradients (shaded rows) and partial sections (white rows), including range and number of analyzed latitudes (N), long-term mean SST minima and maxima across each gradient, average gradient strength and estimated change in gradient strength from 1982–2012 (%).

	Bordering ocean	Gradient	Latitude range^1^	N	Max/min SST (°C)	Mean (s.e.) gradient strength (°C °lat^−1^)	30 year change (%)	*p*
**North America**	Pacific	(1) NAm_Pac_	27–60°N	33	22.5/5.1	**−0.32 (0.02)**	**−8.1**	0.041*
	Atlantic	(2) NAm_Atl_	26–60°N	34	29.7/−1.8	**−0.91 (0.03)**	**−9.6**	<0.001*
		NAm_Atl(1)_	33–39°N	6	28.8/0.1	**−2.55 (0.21)**	**−13.4**	0.001*
		NAm_Atl(2)_	40–57°N	17	23.5/−1.8	−0.76 (0.03)	−4.1	0.301
**South America**	Pacific	(3) SAm_Pac_	8°N–55°S	63	29.0/5.2	−0.29 (0.01)	*+0.7*	*0.820*
		SAm_Pac(1)_	3°N–8°S	11	28.4/16.6	**−0.82 (0.04)**	***+21.9***	0.016*
	Atlantic	(4) SAm_Atl_	8°N–55°S	63	29.6/4.6	**−0.37 (0.02)**	***+3.3***	0.007*
		SAm_Atl(1)_	17–55°S	38	28.4/4.6	−0.55 (0.01)	*+2.3*	*0.200*
**Europe**	Atlantic	(5) Eu_Atl_	37–70°N	32	22.3/3.2	−0.34 (0.01)	−5.3	0.057
**Asia**	Pacific	(6) As_Pac_	22–61°N	39	29.4/−1.7	−0.60 (0.02)	−3.2	0.086
		As_Pac(1)_	22–55°N	33	29.4/−1.7	−0.68 (0.01)	−3.2	0.051
**Africa**	Atlantic	(7) NAf_Atl_	5–35°N	30	29.6/16.3	−0.36 (0.02)	*+5.5*	*0.059*
		NAf_Atl(1)_	7–23°N	16	29.5/17.6	−0.57 (0.02)	−0.5	0.865
		(8) SAf_Atl_	1°N–34°S	35	29.5/13.1	**−0.42 (0.03)**	***+6.2***	0.030*
		SAf_Atl(1)_	12–23°S	11	29.3/13.1	**−0.98 (0.06)**	***+8.5***	0.042*
	Indian Ocean	(9) Af_Ind_	23°N–34°S	53	30.4/19.2			
		Af_Ind(1)_	15–34°S	19	29.9/19.2	**−0.28 (0.02)**	**−7.0**	0.022*
**Australia**	Indian Ocean	(10) AuInd	12–35°S	23	31.4/16.8	−0.46 (0.01)	−5.7	0.226
	Pacific	(11) AuPac	11–39°S	28	29.8/12.3	−0.35 (0.03)	−4.6	0.173

Significant changes (*p*<0.05) are in bold, weakening and strengthening gradients are in normal and italicized fonts, respectively.

1 latitudes in the dataset are given as cell mid-points; hence a latitudinal range of e.g. 27–60°N includes 33 coastal cells from 27.5–59.5°N.

**Figure 2:**
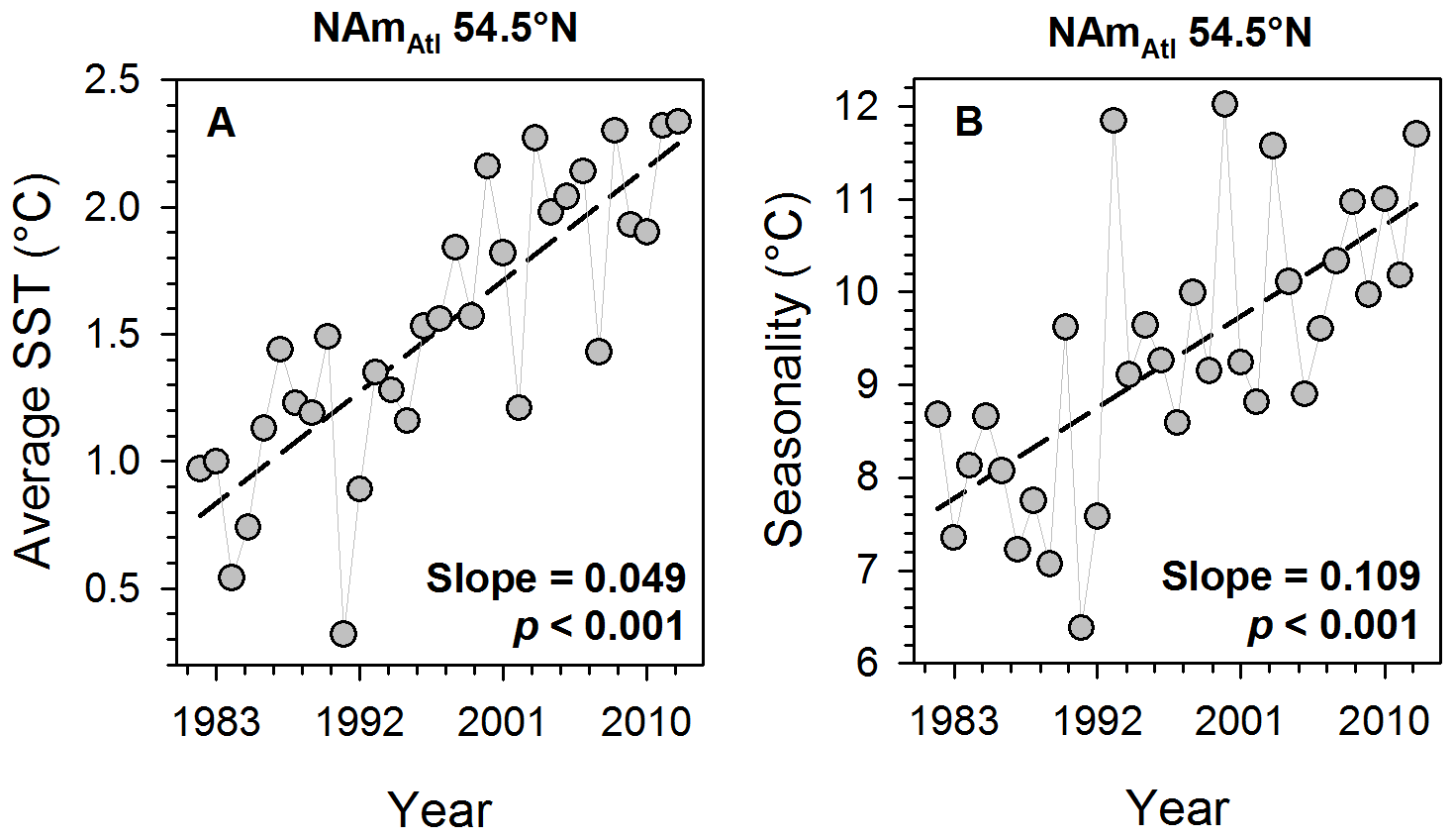
Example of the two linear regressions performed at each latitude, as shown here for 54.5°N of the North American Atlantic gradient (NAm_Atl_). A: annual SST against year B: seasonality against year.

**Figure 3:**
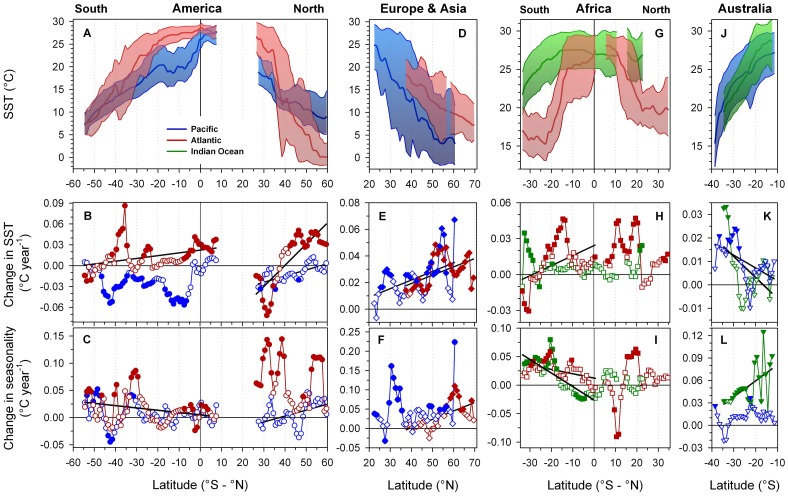
Spatio-temporal temporal patterns of the world's major coastal latitudinal temperature gradients. Upper panel row (A, D, G, J): latitudinal patterns in long-term average SST (thick line), average SST minima and maxima (thin lines) and seasonality (shaded area). Middle panel row (B, E, H, K): latitudinal patterns in 31 year SST trends, with positive and negative slopes indicating warming and cooling, respectively, at specific latitudes.Bottom panel row (C, F, I, L): latitudinal patterns in 31 year trends in seasonality (SST_max_-SST_min_). Empty and filled symbols depict non-significant and significant linear regressions, respectively (*p*<0.05). Significant (*p*<0.05) latitudinal trends in middle and bottom panel rows are indicated by solid regression lines (black). Atlantic Ocean gradients: red lines, shades and symbols; Pacific Ocean gradients: blue lines, shades and symbols; Indian Ocean gradients: green lines, shades and symbols. Positive and negative latitudes refer to the northern and southern hemisphere respectively.

Next we examined two ecologically relevant questions separately for each latitude and gradient: (1) did it get warmer, and (2) did seasons become more extreme over the past 31 years? For each latitude, thus, we linearly regressed (1) mean annual SST and (2) seasonality (annual SST_max-min_) against year (Fig.2). In a second step, the slopes and *p*-values of these regressions were plotted against latitude to identify potential areas of significant (*p*<0.05) warming or cooling within each gradient (Fig.3). We then correlated the latitude-specific regression slopes of both variables to latitude in order to evaluate the hypotheses that higher latitudes have warmed more and increased more in seasonality relative to lower latitudes of a gradient (Fig.3).

**Figure 4:**
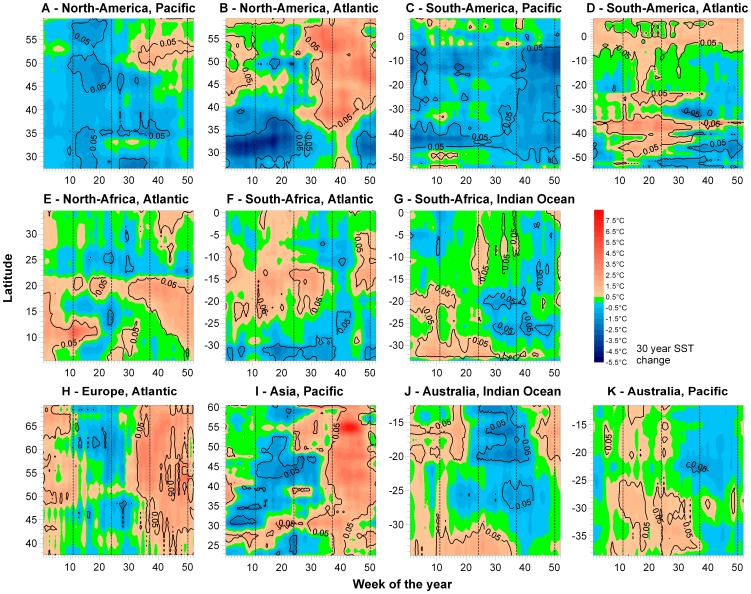
Seasonal decomposition of decadal SST trends in coastal latitudinal temperature gradients. Contours represent the absolute predicted change in SST for each latitude and week, derived by multiplying the linear regression slope *a* by 31 years (SST*_ijk_*  = a*year +*b* with SST of gradient *i*, latitude *j* and week *k*). Isolines enclose areas of regressions with *p*<0.05 (overlaid contour). Dashed lines separate the 52 weeks (wks) into spring, summer, fall, and winter seasons in the northern (NH) and southern hemisphere (SH). Wks 12–24: spring_NH_/fall_SH_, wks 25–37: summer_NH_/winter_SH_, wks 38–50: fall_NH_/spring_SH_, wks 51–11: winter_NH_/summer_SH_.

Last, we examined how changes in average gradients over the past three decades (i.e., based on annual mean SST) might have resulted from seasonal changes in temperature patterns. For example, if a range of latitudes showed a consistent increase (or decrease) in mean annual SST, we asked whether this trend could have been the due to warming (or cooling) only during some seasons of the year. We therefore conducted separate linear regressions of SST on year for each gradient, latitude, and week (433 latitudes ×52 weeks  = 22,516 regressions) and used the slopes and their *p*-values to construct contour plots of predicted latitude- and week-specific SST changes over the past 31 years (contours derived by Kriging, grid spacing for *latitude*, *week*  = 0.5, Golden Software Surfer® V.8, Fig.4). All regression analyses were conducted using SPSS® statistical software (V19 IBM Inc.) and used unadjusted significance levels of *p* = 0.05. This is justifiable by our study's broad focus on consistent patterns over large spatial and temporal scales, which are unaffected by 5% of type I errors [i.e., chance detection of significant relationships, 28]. We assessed, however, the level of temporal autocorrelation in our time-series by calculating the lag-1 autocorrelation coefficients (SPSS® ACF procedure) of detrended time series of (i) gradient strength, latitude-specific trends in (ii) average SST, (iii) seasonality and (iv) weekly SSTs. Time series of gradient strength were not statistically significantly autocorrelated (*p*>0.05), while SST and seasonality time-series showed low levels of temporal autocorrelation (*p*<0.05 for 12% and 5% of the 433 cells, respectively) that did not necessitate a correction procedure.

## Results

### Average gradient characteristics

We found that average slopes vary more than 3-fold among the world's coastal latitudinal temperature gradients (Fig.3, Table 1). The steepest CLTGs are found along the east coasts of the North-American (NAmAtl  =  −0.91°C lat^−1^) and Asian continents (As_Pac_  =  −0.60°C lat^−1^), while the weakest CLTGs occur along the South-African Indian Ocean (Af_Ind(1)_  =  −0.28°C lat^−1^) and the South- and North-American Pacific coasts (−0.29°C lat^−1^ and −0.32°C lat^−1^, respectively). CLTGs of intermediate strength include the European, South-American and South-African Atlantic gradients (−0.34°C lat^−1^, −0.37°C lat^−1^ and −0.42°C lat^−1^, respectively), as well as the Australian Indian Ocean gradient (−0.46°C lat^−1^). Some CLTGs could reasonably be described by linear relationships across their entire latitudinal range (NAmPac, EuAtl, AuInd, AuPac), but most contained regions where the latitudinal SST decline differed notably from the overall average (Fig.3). Along the NAmAtl, for example, SST decreases with −2.55°C lat^−1^ between 33–39°N (by far the steepest CLTG in the world), which is followed to the north by a rate of −0.76°C lat^−1^ (40–57°N). Similarly, sections of the SAmPac and the SAfAtl gradients have slopes twice as strong as their entire range average (SAmPac(1) 3°N-8°S  =  −0.82°C lat^−1^; SAfAtl(1) 12-23°S  =  −0.98°C lat^−1^). Furthermore, the partial SAmPac(1) comprises the world's strongest thermal decline in tropical latitudes (Table 1). In contrast, average SST along the African east coast (AfInd) is almost invariant along a more than 4,000 km long stretch of tropical coastline (SST23_°_N-15_°_S  = 26.0−27.6°C), followed southward by the weak AfInd(1) gradient (15–34°S: −0.28°C lat^−1^). Thus, partial CLTGs are even more variable than entire CLTGs and span almost a magnitude of slope differences (Table 1, Fig.3).

Average seasonality (SST_max-min_) also varies strongly between CLTGsand latitudes (Fig.3). Lowest values generally occur in tropical and subpolar regions (<8°C), while seasonality maxima are located at subtropical and temperate latitudes (25–55°∶10–26°C), with particularly high values in northern hemisphere gradients (NAm_Atl_  = 24.4°C_38.5°N_; As_Pac_  = 25.6°C_39.5°N_). The seasonality peak of the Eu_Atl_ gradient occurs at comparatively high latitudes (15.9°C_53.5°N_), whereas the NAm_Pac_ gradient shows no peak at all but a depression at mid-latitudes (4.1°C_39.5°N_) consistent with the Californian coastal upwelling system. Similar seasonality depressions are also apparent along two other major coastal upwelling areas, i.e., Canary (NAf_Atl_) and Benguela coastal upwelling regions (SAf_Atl_)(Fig.3).

### Spatio-temporal changes in gradient strength & seasonality

Temporal SST trends differed markedly between gradients, latitudes, and seasons of the year, thereby causing complex decadal changes in most CLTGs (Table 1, Fig.3). Out of 11 CLTGs, seven have weakened over the study period (i.e., their absolute latitudinal SST slopes decreased), while four have become stronger (i.e., slopes increased). All northern hemisphere CLTGs except the NAf_Atl_, have decreased in strength by 3–10%, in particular the North-American Atlantic and Pacific gradients (NAm_Atl_: −9.6%, *p*<0.001; NAm_Pac_: −8.1%, *p* = 0.041), the European Atlantic gradient (Eu_Atl_: −5.3%, *p* = 0.057), and the Asian Pacific gradient (As_Pac_: −3.2%, *p* = 0.086). In the southern hemisphere, both Australian gradients did not show statistically significant changes (Au_Ind_: −5.7%, *p* = 0.226 Au_Pac_: −4.6%, *p* = 0.173), whereas the South-African Indian Ocean gradient has lost 7% of its strength over the past 31 years (Af_Ind(1)_ *p* = 0.022). In contrast, significant increases in gradient strength were detected for the South-African and South-American Atlantic gradients (SAf_Atl_: +6.2%, *p* = 0.030; SAm_Atl_: +3.3%, *p* = 0.007). The steep latitudinal decline in tropical SSTs along the South-American Pacific gradient has strengthened further over the past three decades (SAm_Pac(1)_: +22%, *p* = 0.016).

Overall, two thirds of the examined 433 coastal cells showed positive SST trends over time (n = 286), approximately half of which were statistically significant (*p*<0.05, n = 141), while 147 cells had negative SST trends, 55 of which were significant (Fig.3). Regression slopes ranged from −0.07°C year^−1^ (NAm_Atl__31.5°N_) to +0.09°C year^−1^ (SAm_Atl 35.5°S_) corresponding to a 31 year change in local SST of −2.21°C to +2.67°C, respectively. Similarly, trends towards more extreme seasons (i.e., increasing annual SST_max_-SST_min_) were found at more than three quarters of all latitudes (n = 331, *p*<0.05 for 113 cells).

#### AMERICA (Figs.3A–C, 4A–D

Most cells of the ***North-American Pacific gradient*** (NAm_Pac_) showed weak cooling trends (n = 27, *p*<0.05 for 4 cells) with the degree of cooling being latitude-dependent. Maximum cooling was observed in the gradient's lower latitudes, thereby causing a significant decrease in gradient strength over the study period (Fig.3B, Table 1). Seasonality trends, albeit insignificant, changed significantly with increasing latitude (Fig.3C). The seasonal decomposition of the average SST trends revealed that high latitudes (49–55°N) have actually warmed significantly throughout the second half of the year (weeks 35–52), but this effect has been partially offset by cooling trends affecting the late boreal winter to early summer weeks (Fig. 4A). Of the ***North-American Atlantic gradient***'s (NAm_Atl_) 34 coastal cells, 22 showed SST increases (*p*<0.05 for 18 cells) and 12 showed SST decreases over the study period (*p*<0.05 for 9 cells). Warming predominantly occurred at high latitudes north of 42°N (42–60°N, +0.8 to +1.6°C in 31 years), whereas coastal cooling affected all lower latitudes south of 38°N (Florida to Cape Hatteras 26–35°N, −0.5 to −2.2°C in 31 years), therefore causing a 10% decrease in gradient strength over the study period (Fig.3B). Seasonality increased throughout the entire gradient and significantly from north to south (Fig.3C). The seasonal decomposition of SST trends revealed that low latitudes (26–35°N) cooled primarily because boreal winter and spring temperatures decreased strongly, whereas fall temperatures trended slightly upward. Conversely, high latitude warming along the NAm_Atl_ (>42°N) affected the late boreal summer, fall and winter weeks, but not those in spring (Fig.4B). The ***South-American Pacific gradient*** (SAm_Pac_) encompassed 63 coastal cells, of which only 10 had positive, non-significant SST trends, whereas 53 latitudes showed pronounced cooling (*p*<0.05 for 34 cells) at maximum rates of approximately −0.06°C year^−1^ (Fig. 3B). These cooling trends occurred consistently over a large contiguous coastline between 6–47°S (cooling rates of −0.3 to −1.7°C in 31 years, Fig.3C), and resulted from relatively uniform SST decreases throughout the year with the strongest changes seen in austral spring and summer (i.e., weeks 1–6, 35–52, Fig. 4C). However, linear analyses cannot detect potential changes SST patterns due to cyclical changes in El Nino strength and frequency. Neither SST nor seasonality trends showed significant correlations to latitude along the SAm_Pac_ (Fig.3B,C). In contrast, 46 out of 63 cells of the ***South-American Atlantic gradient*** (SAm_Atl_) showed warming trends (*p*<0.05 for 27 cells). Significant warming has occurred along two main clusters located between subtropical 34–41°S and equatorial 8°N–9°S latitudes, while coastal cells south of 45°S showed no or weak negative SST trends, explaining the weak but significant strengthening of the SAm_Atl_ over the study period (Table 1, Fig. 3B). Warming around the equatorial latitudes was caused by uniform trends across all seasons (Fig. 4D), whereas most latitudes south of 24°S showed increased seasonality (Fig. 3C) caused by warming patterns during weeks 5–35 (austral fall-winter) but cooling trends later in the year (austral spring-summer, Fig. 4D).

#### EURASIA (Figs.3D–F, 4H,I)

All of the 32 coastal cells of the ***European Atlantic gradient*** (Eu_Atl_) showed warming trends over the study period (*p*<0.05 for 29 cells). SST trends were significantly correlated to latitude; lower latitudes warmed about half as fast as higher latitudes (mean SST change 37–49°N =  +0.015°C year^−1^, 49–70°N =  +0.035°C year^−1^, Fig.3E), resulting in a loss of gradient strength. Seasonality has increased over the past 31 years mostly at the high latitudes of the Eu_Atl_ gradient (Fig.3F). These trends were attributable to seasonal differences in decadal SST change across the latitudinal range, with warming confined to late boreal summer, fall and winter weeks, partially offset by cooling trends during boreal spring and early summer (Fig.4H). Along the ***Asian Pacific gradient*** (As_Pac_), 38 out of 39 coastal cells exhibited positive SST trends over the study period (*p*<0.05 for 21 cells). Similar to the Eu_Atl_ gradient, warming trends along the As_Pac_ gradient strengthened significantly with increasing latitude (Fig.3E). This, however, was mainly due to four latitudes that have warmed much faster (+0.06°C year^−1^ for 53–56°N and 60–61°N) than all others (∼ +0.02°C year^−1^). Seasonality increased throughout the entire latitudinal range, but significantly mainly between 29–35°N and 53–61°N (Fig.3F) due to seasonal SST dynamics comparable to the Eu_Atl_ and NAm_Atl_ gradients, i.e., warming largely in boreal fall and early winter weeks, being partially offset by SST decreases throughout late boreal winter, spring and summer (Fig.4I).

#### AFRICA (Figs.3G–I, 4E–G)

The ***African Indian Ocean gradient*** (Af_Ind_) had 53 coastal cells, the majority of which showed weak and insignificant warming trends (n = 40, *p*<0.05 for 8 cells). However, the southernmost latitudes (28–33°S) warmed substantially over the past 30 years and also showed trends towards more extreme seasons (Fig.3H,I). The latter was a consequence of SST increases during the first half of the year (weeks 1–30, late austral summer to early winter) but no or weak negative SST trends during the second half of the year (Fig.4G). The ***South-African Atlantic gradient*** (SAf_Atl_) was comprised of 35 cells that showed both significant warming and cooling patterns. The majority of latitudes experienced warming (n = 26, *p*<0.05 for 11 cells), which was most pronounced between 12–23°S and coincided with the steepest section of the SAf_Atl_ gradient (Fig.3G,H). Significant cooling trends, however, were observed at the gradients high latitude end (31–34°S, i.e., south-western tip of Africa), thus resulting in an overall strengthening of the SAf_Atl_ gradient. Seasonality changes were largely insignificant, with a few notable exceptions, including south of 20°S (Fig.3I). Warming and cooling trends were mostly uniform across seasons (Fig.4F). The 30 coastal cells of the ***North-African Atlantic gradient*** (NAf_Atl_) showed mostly warming trends over the study period (n = 25, *p*<0.05 for 17 cells). Tropical latitudes saw greater SST increases than subtropical latitudes, thus explaining the overall strengthening of the NAf_Atl_ gradient (+5.5%). Similar to the SAf_Atl,_ the strongest SST trends occurred where the gradient was steepest, i.e., between 7–23°N Fig3H. Seasonality increased significantly between 15–21°N, but decreased between 9–12°N as a consequence of heterogeneous seasonal warming patterns (Figs 3I, 4E).

#### AUSTRALIA (Figs.3J–L, 4J,K)

The ***Australian Indian Ocean gradient*** (Au_Ind_) consisted of 23 coastal latitudes, of which 16 showed positive SST trends over the study period (*p*<0.05 for 3 cells). Significant warming was restricted to the highest three latitudes (Fig.3K). Increased seasonality was observed throughout the entire latitudinal range of the Au_Ind_ gradient, with stronger trends at lower latitudes (Fig.3L). This could be attributed to the seasonal decomposition of SST changes, revealing that low latitudes (11–21°S) warmed mainly during the first 20 and last 10 weeks of the year (late austral spring, summer, fall), but exhibited opposite SST trends during austral winter and early spring weeks (weeks 20–42, Fig.4J). In contrast, the highest Au_Ind_ latitudes south of 30°S primarily warmed in austral fall and winter (weeks 10–37). The ***Australian Pacific gradient*** (Au_Pac_) encompassed 28 latitudes, most of which showed warming trends (n = 25 *p*<0.05 for 7 cells) that were strongest at the gradients southern end (Fig.3K), thus slightly weakening the AuPac. Seasonality trends were non-significant, but consistently positive throughout the gradient range (Fig.3L). Seasonal decomposition indicated that significant warming affected all latitudes but only during austral fall and winter weeks (weeks 10–35, Fig.4K).

## Discussion

Our study characterized how SST along the world's major coastlines changes with latitude, and how these large-scale thermal clines have themselves changed over the past three decades. This is of interest, because coastal and continental shelf regions are the most diverse, productive, and economically important parts of the ocean [1], and because temperature is the most important abiotic factor influencing marine life from the cell to the ecosystem level [4], [5]. Hence, coastal ecological shifts in both space (i.e., latitude) and time (i.e., decade) are likely driven in part by the kind of latitudinal temperature patterns described here. We found considerable variation in gradient strength and seasonality between the world's coastal regions, while documenting complex and often opposing decadal trends in these metrics, consistent with a body of related previous works that have detailed the regional variability of unfolding global climate change [6], [8], [19], [21], [29]–[32].

SST was deemed a suitable proxy for average thermal conditions in shallow coastal areas. In addition, SST can be measured remotely and is therefore available at high data density and consistency over long periods of time [26], [27]. The land-sea mask of the data set facilitated an objective algorithm for selecting coastal cells from the global grid, with the acknowledged trade-off of narrowing coastal representation with increasing latitude (e.g., one degree of longitude at 0° and 60° is 111.3 and 55.8 km wide, respectively). Our approach was to apply simple regression analyses over broad spatial and temporal scales to yield the desired general picture of gradients and their changes over past 30 years, thereby ignoring analyses of potential non-linear or cyclic patterns in global climate (e.g., ENSO, AMO).

### Patterns & changes of coastal latitudinal temperature gradients

Latitudinal temperature patterns along coastlines are much more heterogeneous than in the open ocean [8], [31], [32], likely because of regional differences in well-understood oceanographic and climatic features such as alongshore surface currents, upwelling, thermocline depth, wind patterns, and cloudiness [7]. The world's strongest coastal gradients (i.e., NAm_Atl_ and As_Pac_), for example, occur along the western boundaries of the Northern Hemisphere Atlantic and Pacific Oceans, where opposing warm and cold alongshore currents transport subarctic waters south- and tropical waters northward (NAm_Atl_: Florida/Labrador Currents, As_Pac_: Kuroshio/Oyashio Currents). The European gradient's (Eu_Atl_) elevated thermal profile [7], [33], with coastal latitudes being on average 5–10°C warmer than the same latitudes along the NAm_Atl_ or As_Pac_, is consistent with the influence of the North-Atlantic Drift, i.e., the warm continuation of the Gulf Stream past its main branching point. Similarly, the world's weakest gradient was found along the South-African east coast (Af_Ind(1)_ −0.28°C lat^−1^), where another western boundary current - the warm Agulhas Current - transports heat southward [34]. Coastal upwelling, on the other hand, appears to be recognizable by distinct temperature depressions (‘troughs’) that cause substantially different regional gradient slopes. This signature pattern appeared in all four major coastal upwelling regions (Fig.3), i.e., (i) the California Current upwelling system (NAm_Pac_), (ii) the Benguela Current upwelling system (SAf_Atl_), (iii) the Canary Current upwelling region (NAf_Atl_), and (iv) Humboldt Current upwelling system off Peru and Chile (SAm_Pac_). In the latter, the observed depression in coastal temperatures (5–17°S) result in a uniquely steep tropical SST decline with latitude (SAm_Pac(1) 3°N-8°S_  =  −0.82°C lat^−1^), which sets a potentially great natural contrast for large-scale ecological comparisons.

Temporal trends in CLTG patterns likely represent the complex net result of changes in atmospheric and oceanographic features [1], [35], both of which are expected to differ between regions. However, we found that the NAm_Atl_, Eu_Atl_ and As_Pac_ gradients share a common Northern Hemisphere warming pattern [21], [30] that involves intensified warming at high compared to lower latitudes, generally as a consequence of precipitous SST increases during fall and winter (wks 35–11) that are partially offset by cooling trends in spring and summer (wks 12–34) [6]. This has caused the weakening in overall gradient strengths while increasing seasonality across latitudes. The latter is consistent with Lima & Wethey's [6] finding that the incidence of extreme SSTs (warm and cold) has increased in most coastal waters. However, common patterns may be evoked by different mechanisms [21], such as increased heat transport at high latitudes, stronger stratification due to warmer freshwater run-off [21] or changing ocean currents [36], [37]. For example, the NAm_Atl_ weakened not only because of high latitude warming, but also because of strong cooling trends in coastal waters between 26–35°N [6]. SSTs in coastal waters between the tip of Florida (25°N) and Cape Hatteras (35°N) are influenced by the warm Florida Current, i.e., the beginning of the Gulf Stream [38]. Cooling of this coastal section is intriguing [39], given that it might signal an overall decrease in the Florida Current heat transport or a progressive diversion of warm Florida Current waters from the coast, both of which have been suggested by Ezer et al. [37] in a recent analysis. These SST changes have been substantial and therefore likely had profound ecological consequences for this region. Another example can be found along the Australian East coast (Au_Pac_), where the warm East Australian Current has been shown to extend further and further southward [40], which is consistent with the significant warming trends observed between 36–39°S along the Au_Pac_ (mainly an austral fall and winter phenomenon) [6].

In general, SST trends at individual latitudes were consistent with those reported in studies of regional coastal temperature change [21]. For example, Nixon et al.'s [41] analysis of a 117-year SST record from the pier at Woods Hole, Cape Cod (located at ∼41.5°N on the North-American Atlantic coast) showed average warming rates of 0.04°C year^−1^ between 1970–2002, which is comparable to rates estimated here (NAm_Atl, 41.5°N_  = 0.02°C year^−1^ and NAm_Atl, 42.5°N_  = 0.03°C year^−1^). Similarly, Gomez-Gesteira et al. [7] found that SST in coastal European waters between 37–48°N increased at annual rates of 0.012 to 0.035°C year^−1^ (1985–2005), which is consistent with the present estimates of 0.014 (Eu_Atl,__37.5°N_) to 0.034°C year^−1^ (Eu_Atl,__49.5°N_).

Coastal upwelling has been predicted to intensify as a result of increasing wind-stress linked to global climate change . [1], [35], [42], [ but see 43]. Our analyses support this prediction by identifying a second remarkable pattern shared by some coastal latitudinal SST gradients: cooling [6]. This has been most evident along the South-American Pacific coast, where all latitudes between 6–47°S but particularly those between 6–14°S have consistently cooled (average of −1.58°C in 31 years), which is spatially consistent with the Humboldt Current upwelling system [44]. In addition, cooling affected all seasons of the year, but was most intense during austral spring and summer (wks 35–5). Hence, the substantial strengthening (+22%) of the partial SAm_Pac(1)_ gradient, i.e., the unique decline in tropical SSTs along the South-American Pacific coast, may have largely been a consequence of upwelling intensification [21]. Similar patterns, albeit weaker, were also detected for the region of the NAm_Pac_ gradient corresponding to the California Current upwelling system, for which upwelling has been examined in more detail [21], [45], [46]. Intensification of the South African Benguela upwelling system, as suggested by Santos et al. [32] is consistent with the cooling patterns we detected at the southern edge of the SAf_Atl_ gradient. It may also explain why coastal latitudes of the Canary Upwelling system (22–30°N) showed no significant SST trends, contrary to substantial warming observed at all other coastal cells of the NAf_Atl_ gradient [6], [31]. Conversely, several studies have detected a weakening of upwelling intensity along the western Iberian coast [47], which corresponds to small but significant warming trends along the lower latitudes of the Eu_Atl_ gradient in this study. In general, decade-scale cooling had likely as severe, if opposite, ecological consequences for marine life as decadal warming.

We conclude that the complex and regionally heterogeneous patterns imply that direct heat transfer from the atmosphere to ocean (i.e., global warming) cannot by itself explain the observed changes in CLTGs. Regional scale, wind-driven, indirect effects, via heat fluxes and upwelling, must drive changes in both the magnitude and seasonality of coastal SST gradients.

### Ecological implications

Given temperature's fundamental role in governing marine life, long-term changes in CLTGs will likely precipitate shifts in ecological gradients. Adaptive latitudinal variations in intra-specific morphological, physiological and life history traits are well documented in marine taxa [reviewed in 12] and encompass traits such as body size [10], [11], growth and development rates [48]–[50], predator vulnerability [51], life span [52], or vertebral number [16], [17]. For example, adapting to decreasing temperatures with latitude, many estuarine fish populations from high latitudes have evolved higher genetic growth capacities than their lower latitude conspecifics, a form of genotype × environment interaction known as countergradient variation [53]–[55]. Baumann and Conover [56] found that the strength of countergradient growth variation in silverside fishes from the North-American Pacific versus Atlantic coasts mimicked the strength of their contrasting latitudinal SST gradients. Similarly, increases in vertebral number with latitude, a form of co-gradient variation [12], is twice as strong in silverside fishes along the North-American Atlantic (strong CLTG) versus the Pacific coast [weak CLTG, 57]. Hence, latitudinal temperature gradients likely determine genotype*environment interactions in coastal systems, and shifts e.g., towards weaker gradients may precipitate weaker genetic gradients by reducing the amount of genetic variance between populations.

These adaptive trait gradients along CLTGs may comprise useful spatial analogies for studying climate change adaptation [12], [56]. How organisms will adapt to global climate change is uncertain, partly because of difficulties besetting the methods to study it (e.g., duration, replication, plastic vs. genetic responses). In contrast, adaptation principles can be rigorously examined across spatial climate gradients of different strength, such as the world's CLTGs. If countergradient variation, for example, is as ubiquitous an adaptation principle in time as it is in space, global warming may not lead to oft-anticipated increases in observed growth rates among ectotherms [1], [12]. Instead, genetic decreases in growth capacity could offset the plastic, temperature-driven growth increases, analogous to the local growth adaptation in high versus low latitude fish populations [56].

CLTGs may also underlie the ubiquitous decline in species diversity from low to high latitudes [9], [58], [59]. Whether coastal diversity actually scales with the strength of the underlying temperature gradient has received less attention, but could be effectively tested using the full and partial thermal slopes described here. Paleo-biological analyses indicated that diversity-temperature relationships in planktic foraminifers have been surprisingly stable over the past three million years, suggesting that gradients in species richness rapidly reorganize in response to climatic changes [58]. Hence, weakening thermal gradients due to large-scale latitudinal temperature shifts could precipitate weaker diversity gradients, by increasing diversity at high latitudes and/or decreasing diversity at low latitudes [e.g., 60].

One already widely observed biotic reaction to global climate change [60]–[64], is the shift in geographic ranges in marine species, with the vast majority comprising poleward movements [e.g., 61: 75% of 129 marine species]. This is consistent with our observation of weakening coastal latitudinal temperature gradients, not only because high latitude warming increases potential habitat for lower latitude organisms, but also because decreases in thermal gradient strength would make habitats more similar across latitudes and thus even more suitable for some intermediately located genotypes. In the North Atlantic Ocean, for example, where both coastal temperature gradients (Eu_Atl_, NAm_Atl_) have weakened substantially, range shifts over the past decades are particularly evident, e.g., in the fish assemblages in the North Sea [65] and along the North-American continental shelf [66]. On the other hand, the considerable variability in geographic trends among regions of the world is consistent with the substantial differences in coastal thermal gradients we have described here. While focusing on temperature alone is likely too simplistic to predict shifting species distributions, ‘bioclimate-envelope’ approaches are still useful on large spatial and temporal scales [67], [68] and may benefit from explicitly incorporating coastal latitudinal temperature gradients.

Lastly, shifts in coastal temperature patterns like the observed trends towards more extreme seasons may affect the connectivity between coastal marine populations. More extreme winters could increase overwinter mortalities in some organisms, while more extreme summers could both extend or curtail reproductive and growing seasons in others, thereby potentially turning continuous species distributions into more mosaic ones. Along the Australian Indian Ocean coast (Au_Ind_), for example, seasonality increased significantly along the entire gradient, a trend that may challenge stenothermal tropical organisms (e.g., some corals or reef fish). Reduced connectivity may also result from opposing temperature trends between adjacent coastal regions. At the southern tip of the African continent, for example, the warm African Indian Ocean gradient and the bordering cold South-African Atlantic gradient have exhibited divergent trends over the past decades. Our analyses indicated significant warming on the Indian Ocean side (Af_Ind_: SST_32.5°S_  = 22.3°C_1982_→23.3°C_2012_) but cooling on the Atlantic Ocean side (SAf_Atl_: SST_32.5°S_  = 17.0°C_1982_→16.3°C_2012_), thereby exacerbating the temperature differential that migrating organisms must cross by more than 25% (5.3 to 7.0°C).

### Conclusions

We found that the rate of latitudinal temperature change along the world's major coastlines varies more than threefold and has – in seven of 11 cases – decreased over the past three decades. SST and seasonality have increased in the majority of coastal cells, with the greatest changes observed in Northern Hemisphere gradients, but opposite trends, i.e., coastal cooling, have occurred particularly at upwelling influenced coastlines. As expected, decadal changes in gradient strength, latitude-specific SST and seasonality were found to be highly heterogeneous between regions, yet consistent with regionally differing consequences of global climate change. We suggest that past and future ecological changes in coastal marine ecosystems are linked to these regionally diverse CLTG patterns and that quantifying them is useful for future meta-analyses of large-scale ecological and thermal gradients. This would improve the current understanding about adaptive responses of marine life to climate gradients in space and, by inference, time. Valuable next steps include the identification of non-linear and cyclic SST trends, backward extension of the SST dataset, and forecasts of thermal gradient evolution under different IPCC scenarios [3] in general circulation climate models.
